# Have we forgotten something when caring for patients for surgery?

**DOI:** 10.3389/fmed.2022.952893

**Published:** 2022-07-28

**Authors:** Zhiyi Zuo

**Affiliations:** Department of Anesthesiology, University of Virginia, Charlottesville, VA, United States

**Keywords:** family members, neurobehavioral status, surgery, anxiety, interventions

## Introduction

An astonishing number of patients will have surgery each year in the USA and the world. Among them, 20 to 40% of patients will suffer from cognitive dysfunction during the hospitalization and about 10% of elderly patients will have cognitive dysfunction months later (1, 2). Similarly, 10 to 60% of elderly patients will develop postoperative delirium (3). Patients suffering from postoperative delirium or cognitive dysfunction have a poor outcome including longer hospitalization and a higher rate of mortality and leaving the job market (1, 2). Thus, postoperative delirium and cognitive dysfunction are very significant issues.

Undoubtedly, a cognition uncompromised patient for surgery will be anxious during the perioperative period. Over-anxiety and other unhealthy neuropsychological activities may negatively affect the outcome of patients. For example, patients with depression symptoms have a higher incidence of postoperative delirium (4). Also, patients with postoperative cognitive dysfunction tend to have higher Beck depression inventory scores (*P* = 0.089) (2). However, the neuropsychological status of patients is not evaluated before the surgery in current practice. This evaluation is not performed in the majority of studies aiming to determine the outcome of surgical patients. Understandably, measures to improve neuropsychological status during the perioperative period have not been routinely applied to patients. However, the importance of the pre-surgery screening for postoperative delirium and cognitive dysfunction has been emphasized by an expert panel from the American Society of Anesthesiologists. The interventions recommended by this panel are general measures including reducing the use of medications that may contribute to the development of these postoperative cognitive disorders and providing a familiar environment as much as possible (5). These efforts are the initial steps to improve perioperative brain health for patients with surgery. In addition, a checklist targeting 8 areas before surgery has been proposed by the “Strong for Surgery” program that is now sponsored by the American College of Surgeons. Among the 8 areas, two areas, screening the risk for delirium and prehabilitation, are important elements for perioperative brain health.

## Potential effects on the relatives of patients with surgery

Obviously, patients for surgery are not in isolation. They have family members and friends. The consequences of surgery on the family members and close friends of surgery patients have been largely unknown. Neurobiologically, behaviors and feelings can be “contagious.” Itch and pain sensation can be transmitted to observers (6, 7). Stress responses are transmitted to subjects that are not exposed to the initial stress stimuli (8). Familiarity is an important factor for the transmission of the behaviors and feelings (7, 8). Interestingly, consolation, a behavior to comfort the injured or distressed individual, occurs in animals. This behavior toward distressed subjects is oxytocin-dependent and involves anterior cingulate cortex in prairie vole (8). The distress was caused by separation, loud tone and foot shocks in that study. Our recent study has shown that consolation occurs from cage-mates toward individuals with surgery in mice. This behavior reduces the anxiety of surgery mice. The interaction between surgery mice and non-surgery mice increases the anxiety of non-surgery mice. The orexin signaling in the paraventricular thalamic nucleus may play a critical role in the consolation and anxious behaviors of non-surgery mice (9). These basic science studies have shown the transmission of behaviors and anxiety among animals.

Humans are highly social and have a much more complex system for communication and interaction. Undoubtedly, family members, especially the close family members of patients for surgery, will be anxious, particularly when the patients will have a major surgery (10). Consolation and care of the family members toward the patients with surgery will likely have a positive effect on the recovery of the patients. Anxiety induces physiological responses and has a detrimental effect on patients with various diseases (11). However, the effects of anxiety and other negative psycho-behaviors of family members on their health are rarely studied. The influence of potential negative interaction between patients and family members in the health of both parties and surgery outcome is not known. Interestingly, non-surgery cage-mates of mice with surgery develop learning and memory impairment, similar to the presentation of mice with surgery. Surgery mice and their non-surgery cage-mates have increased inflammatory cytokines in their brain (12). Although the mechanisms for the impaired learning and memory in the non-surgery cage-mates are not known, neuroinflammation is known to impair learning and memory and is a major pathological process for postoperative delirium and cognitive dysfunction (2, 13). Similarly, a population-based study has shown that spousal caregivers of a patient with incident dementia have a six-fold increase in the hazard for incident dementia later in life compared to others whose spouses are not demented (14). Also, spousal caregivers of patients with dementia have a greater cognitive decline than spouse caregivers of non-dementia patients (15). Anxiety and stress may be contributing factors for the findings in the spouses (14, 15). Thus, reducing anxiety and stress is a possible approach to improve the brain health of these spouses. These studies illustrate an important and largely untouched field in perioperative medicine, the health of patients' family members and close friends, and the effects of their interaction with patients on the outcome of patients after surgery. In supporting the potential of these effects, a recent study has shown that the quality of life of patients with stem cell transplantation is correlated with the anxiety and depression of their family caregivers (16).

## Current practice to reduce anxiety of patients with surgery and their family members

Effective communication and frequent updates on surgery progress are good practices to reduce the anxiety and stress of patients with surgery and their relatives. Excellent support from the social community including friends may prepare the patients for better recovery and family members for less stress. Many of these practices are in place for patients with surgery. Advanced training for perioperative care providers on effective communications and consolation skills will be important elements to improve the wellbeing of the patients for surgery and their family members.

## Proposed measures to improve the outcome of patients with surgery and their family members and relevant discussion

Additional work can be done to better prepare the patients and their family for surgery. It is important to evaluate the patients and family members to identify factors that increase anxiety and stress and to mitigate these factors. Consolation toward the patients with surgery and their family members should be part of the practice of health care providers. More importantly, applying appropriate interventions and programs to improve the neuropsychological status should be incorporated into the perioperative care. Measures to improve brain functions, such as environmental enrichment, should be applied. A multidisciplinary team focusing on reducing anxiety, distress and other negative psycho-behaviors and improving brain functions should be formed for patients with surgery, especially for those at high risks for postoperative neurocognitive disorders. The team members should include the anesthesiologist, surgeon, neuropsychological specialist, and social worker. The team can be named perioperative enhancing brain-health service (PEBS) (Table 1). This practice will be similar to that of the acute pain service that was not provided routinely 30 years ago. Finally, close follow-up of surgery patients and their family conditions after surgery should be performed by PEBS. To achieve the goals of PEBS, PEBS consultation for those patients who will require significant care from the family members after surgery or are at a high risk for developing postoperative neurocognitive disorders should be requested immediately after the decision for surgery has been made to give PEBS enough time to work with the patients and their family members. Preoperative screening, preparations and interventions can be performed in the form of family-based neurocognitive prehabilitation to increase the resiliency of patients and family members to harmful effects on the brain during the perioperative period. A neuropsychological specialist may lead these activities but a close working relationship among the members of PEBS is needed to provide a timely and effective program for patients and family members throughout the perioperative period. These practices are different from the commonly referred prehabilitation that emphasizes interventions for patients before surgery. The success of PEBS service can be measured by not only the decrease in the rates of postoperative delirium and cognitive dysfunction, length of hospitalization, and number of patients returning to work force but also the wellbeing of their family members, such as anxiety and depression levels, number of missing work days and percentage of them returning to their jobs. Considering that more than 50 million patients have surgery annually in the USA, there are more family members who will benefit from these practices. Let us work together to make the perioperative period a less fearful and anxious time for the patients and family members. These practices will maximize the benefit of surgery to the patients, their family members and ultimately to our society.

**Table 1:** Proposed changes in evaluating and preparing patients for surgery.

**Present model**
Patient is evaluated before surgery to optimize cardiac and pulmonary functions for surgery.
Patient may be evaluated by acute pain service for pain management.
**New model**
Patient is evaluated before surgery to optimize cardiac and pulmonary functions for surgery.
Patient is evaluated by PEBS to optimize brain health of patients and family members for surgery.
Patient may be evaluated by acute pain service for pain management.
Patient will be followed up by PEBS for neuropsychological management.
Perioperative care providers have additional training on effective communications and consolation

## Author contributions

The author confirms being the sole contributor of this work and has approved it for publication.

## Funding

This study was supported by the Robert M. Epstein Professorship endowment (to ZZ), University of Virginia, Charlottesville, VA.

## Conflict of interest

The author declares that the research was conducted in the absence of any commercial or financial relationships that could be construed as a potential conflict of interest.

## Publisher's note

All claims expressed in this article are solely those of the authors and do not necessarily represent those of their affiliated organizations, or those of the publisher, the editors and the reviewers. Any product that may be evaluated in this article, or claim that may be made by its manufacturer, is not guaranteed or endorsed by the publisher.
